# Left-Right Asymmetry in the Sea Urchin Embryo: BMP and the Asymmetrical Origins of the Adult

**DOI:** 10.1371/journal.pbio.1001404

**Published:** 2012-10-09

**Authors:** Jacob F. Warner, Deirdre C. Lyons, David R. McClay

**Affiliations:** Department of Biology, Duke University, Durham, North Carolina, United States of America

## Abstract

Bilateral animals, including humans and most metazoans, are not perfectly symmetrical. Some internal structures are distributed asymmetrically to the right or left side. A conserved Nodal and BMP signaling system directs molecular pathways that impart the sidedness to those asymmetric structures. In the sea urchin embryo, one such asymmetrical structure, oddly enough, is the entire adult, which grows out of left sided structures produced in the larva. In a paper just published in *PLOS Biology*, BMP signaling is shown to be necessary early in larval development to initiate the asymmetric specification of one of those left-sided structures, called the left coelomic pouch. This study reports that BMP signaling activates a group of transcription factors asymmetrically in the left coelomic pouch only, which launch the pathway that eventually leads to the formation of the adult that emerges from the larva at metamorphosis.

## Abstract 2

The entire adult sea urchin develops on the left side of the larva. This Primer discusses a new study that reports that BMP signalling activates this process while Nodal signalling inhibits it on the larva's right side.

## Sea Urchin: Indirect Development to Adulthood

The sea urchin is a marine invertebrate deuterostome in the phylum Echinodermata. Most species of sea urchin are indirect developers: the embryo develops first into a swimming larva that feeds on plankton while its adult structures grow inside the larva. At metamorphosis, most larval structures are lost and the adult emerges, settles onto the substrate, and grows to sexual maturity. Superficially, the adult displays pentaradial (five-sided) symmetry, while the larva typically has a bilaterian organization with left-right and dorsal-ventral (aboral-oral) axes.

**Figure 1:**
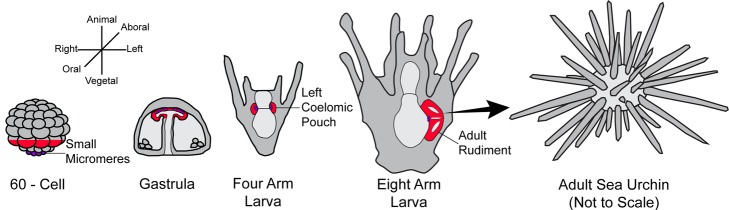
Left-right asymmetry in the sea urchin. During cleavage, mesoderm (red cells at 60-cell stage) and small micromeres (purple cells at vegetal pole) become specified. At the end of gastrulation, progeny of these two cell types contribute to the coelomic pouches (red and purple outpockets seen at the tip of the archenteron). During larval stages, the adult rudiment grows from the left coelomic pouch. After metamorphosis, that rudiment grows to become the adult. Embryonic axes are shown relative to the 60-cell cleavage and gastrula stages. The aboral-oral axis is also known as the dorsal-ventral axis. The animal-vegetal axis is the only axis established prior to fertilization. Oral-aboral axis specification occurs early in cleavage, and left-right axis determination occurs at the late gastrula stage.

In the embryo, two coelomic pouches bud from the anterior end of the foregut, and these structures exhibit left-right asymmetry. Coelomic pouches are mesodermal structures produced by all animals with a body cavity. That future adult body cavity is called a coelom, and its lining originates in the sea urchin from the mesodermal coelomic pouches (CPs). During larval growth in the sea urchin, the left CP becomes modified and produces major components of the rudiment (the term used for the primitive form of the adult) (Figure 1). Mesoderm and primordial germ cells initially contribute to the left coelomic pouch of the embryo, and later, larval ectoderm and endoderm are added to form the rudiment, which grows until it dominates the mass of the larva. Its location on the left side of the larva places special importance on the signals that establish sidedness to the embryo.

## Signals to Left and Right

The left-right asymmetrical signaling system is used by bilaterians to correctly position asymmetric processes and internal organs and tissues including, in vertebrates, deflection of the heart tube, gastrointestinal folds, sites of endodermal organ budding (liver, lung, and pancreas), and others [1]–[4]. Nodal signaling plays a central role in all of these patterning decisions. In 2005, Nodal was discovered to participate in establishing L-R asymmetry in sea urchin embryos as well, but in sea urchins, rather than being expressed on the left side (as it is in vertebrates), Nodal was expressed on the right side, where it was shown to inhibit the right CP from forming the adult rudiment. If Nodal function was inhibited at the time the asymmetry occurred, both the right and left CP produced rudiments [5]. In contrast, if Nodal signaling was forced to operate on both sides, no rudiment was produced [5]. These data established an important asymmetric inhibitory role for Nodal and indicated that the sea urchin shares a conserved relationship with other many other bilaterians, even though details of that symmetry-breaking process vary between species. A key piece of information that was missing in the sea urchin was the identity of positive signals that stimulate the development of the left CP, a necessary component of the adult rudiment.

In a study reported in this issue of *PLOS Biology*, Luo and Su [6] now demonstrate that BMP signal transduction is crucial for the specification and organization of the left CP with no detected activity on the right CP. Antibody detection of a phosphorylated Smad, the transcription factor that responds to the BMP signal, called Smad 1/5/8 in the sea urchin, allowed the investigators to observe which cells responded to the BMP signal, and thus initiate left CP, and eventually rudiment development. They carefully monitored two types of cells that contribute to the left CP. At the end of gastrulation, a group of mesoderm cells initially form the CPs (Figure 1), and these were joined by the progeny of the small micromeres, which moved to both CPs, with the majority of the eight small micromeres generally joining the left CP. To study the sequence of events involved in coelomic pouch formation in response to the BMP signal, Luo and Su monitored a number of transcription factor markers over time. The BMP signaling activated the mesoderm CP cells through Smad 1/5/8 but had no direct effect on the small micromeres. From their data, they then constructed a spatio-temporal profile of the gene expression subdomains to model the early morphogenetic events in CP development. This morphogenetic sequence provides a useful template for future studies of how the rudiment forms. Luo and Su's findings also show that small micromeres, which originate at the vegetal pole of the embryo, and enter both CPs, are gradually lost by apoptosis if they enter the right CP, presumably because of Nodal signaling there. This is truly a fateful asymmetric decision because the small micromeres are thought to become the primordial germ cells of the next generation [7]. Thus if they go left they live, but if they go right they die.

## Evolutionary Depth to Left-Right Patterning

The study adds evolutionary depth to an expanding list of organisms, ranging from snails to vertebrates, in which the Nodal and BMP pathways contribute to the establishment of L-R asymmetry. At the same time, although there is a clear molecular conservation of signals in L-R patterning, the detailed mechanism differs among the deuterostomes and non-deuterostome bilateria. As seen in the sea urchin, for example, Nodal is used on the right side as a patterning signal rather than on the left as it is in vertebrates. Several recent reviews discuss the mechanism of establishing asymmetry and in addition point out missing information and misconceptions about the process [4],[8]. For example, in vertebrates the movement of Nodal toward the left side of the node by beating cilia was an exciting discovery [2], but that mechanism is not universal as different processes operate at the node in other groups of vertebrates. In addition, the causal symmetry-breaking event is not understood in any bilaterian, although there are several potential contributors to the process ranging from calcium signaling and endocytosis to centrosome distribution and cytoskeletal organization [4].

Many questions remain concerning how initial L-R asymmetry is established and how that event eventually causes the asymmetric expression of Nodal and BMP. In the sea urchin embryo, Nodal and BMP are also part of the mechanism that establishes the Dorsal-Ventral (aboral-oral) asymmetry [9], and as is in vertebrates, both of these TGFß signals are involved in mesoderm specification [10],[11]. As a consequence, the discovery of the complete pathway leading to asymmetric left or right side function of Nodal and BMP will be challenging since both signals have multiple roles in early development.

Thus, although much remains to be learned about the sequence of events in establishing L-R asymmetry, the groundwork provided in the Luo and Su article establishes an important signal, a cell lineage, and a morphological sequence of gene expression that directs early rudiment formation. These data will also guide and inform future investigations into somatic and germ cell development.
